# Effect of one and two sessions of antimicrobial photodynamic therapy on clinical and microbial outcomes of non-surgical management of chronic periodontitis: A clinical study

**DOI:** 10.15171/japid.2019.014

**Published:** 2019-12-18

**Authors:** Ardeshir Lafzi, Seyed Masoud Mojahedi, Mahdieh Mirakhori, Maryam Torshabi, Mahdi Kadkhodazadeh, Reza Amid, mohamadjavad karamshahi, Mohammad Arbabi, Hasti Torabi

**Affiliations:** ^1^Department of Periodontics, School of Dentistry, Shahid Beheshti University of Medical Sciences, Tehran, Iran; ^2^Department of Laser, Dental School, Shahid Beheshti University of Medical Sciences, Tehran, Iran; ^3^Dental Research Center, Research Institute of Dental Sciences, school of dentistry, Shahid Beheshti University of Medical Sciences, Tehran, Iran; ^4^Dental Biomaterials Department, School of Dentistry, Shahid Beheshti University of Medical Sciences, Tehran, Iran; ^5^Department of Periodontics, School of Dentistry, Shahid Beheshti University of Medical Sciences, Tehran, Iran; ^6^Department of Periodontics, School of Dentistry, Qazvin University of Medical Sciences, Qazvin, Iran; ^7^DDS, Private Practice, Tehran, Iran

**Keywords:** Chronic periodontitis, non-surgical periodontal therapy, photodynamic therapy, scaling and root planing

## Abstract

**Background:**

This study aimed to compare the effect of one and two sessions of antimicrobial photodynamic therapy (aPDT) as an adjunct to scaling and root planing (SRP) on clinical and microbial parameters in patients with chronic periodontitis.

**Methods:**

This study was conducted on 20 patients. The dental quadrants of patients were randomly assigned to SRP at baseline (group 1), SRP at baseline and one month (group 2), SRP plus aPDT at baseline (group 3) and SRP plus aPDT at baseline and one month (group 4). Probing depth (PD), clinical attachment level (CAL) gain, and bleeding on probing (BoP) were measured at baseline, and one and three months later. F. nucleatum counts were determined by PCR. ANOVA was used for the comparison of these variables between the groups.

**Results:**

In all the groups, PD reduction and CAL gain increased significantly at 1- and 3-month intervals compared to baseline (P=0.001). At three months, the difference in PD between groups 1 and 3 was statistically significant (P=0.014). CAL gain between groups 2 and 4 at one month (P=0.016) and three months (P=0.001) wasstatistically significant. Reduction in F. nucleatum counts was not significant between the four study groups (P>0.05).

**Conclusion:**

A combination of two sessions of aPDT and SRP could improve CAL gain; however, further long-term studies are necessary in this regard.

## Introduction

Although scaling and root planing can cause a significant improvement in many patients with chronic periodontitis, it cannot completely eliminate subgingival calculus and periodontal pathogens.^1,2^ Conventional periodontal treatment cannot provide proper access to distant and hard-to-reach areas, such as the distal surface of molars, furcation areas, concavities, grooves, and deep pockets;^3^ as a result, bacteria might remain on the root surface and compromise the outcome of periodontal therapy.^4^ For this reason, supplemental treatments are required to enhance the efficacy of non-surgical periodontal therapy.^5^ However, considering the gradual improvement in probing depth (PD) and clinical attachment level (CAL) gain as a result of scaling and root planing (SRP), the conventional mechanical debridement remains the gold standard for the long-term success of treatment.^6^

Novel adjunct treatments have been suggested to improve the outcomes of current treatment modalities, such as laser therapy, local and systemic antibiotics, and subgingival placement of chlorhexidine chips.^2^ Among locally used antibiotics, tetracycline, minocycline, and metronidazole, as well as chlorhexidine antimicrobial agents, have yielded the most favorable results.^3^ However, the use of antibiotics is associated with the emergence of antibiotic-resistant species.^4^ Thus, the use of systemic antibiotics must be limited to a specific group of periodontal patients, such as those suffering from a very acute form of disease or a particular microbial profile.^5^

Antimicrobial photodynamic therapy (aPDT) can serve as a suitable alternative to conventional antimicrobial treatments.^6^ In aPDT, a photosensitizer binds to the respective bacterial species and is activated by light at 630‒830-nm wavelength.^7^ After absorption of the light energy, some changes occur in the photosensitizer, and it finally reacts with oxygen and produces free oxygen, superoxide and hydroxyl radicals.^8-10^ aPDT is effective against both antibiotic-resistant and antibiotic-sensitive bacteria, and its repetition cannot cause resistance.^11^ Thus, aPDT has been suggested as an alternative to the inactivation of bacteria within the biofilm.^12-17^

The first report comparing SRP with and without aPDT showed greater improvements in clinical parameters in the SRP plus aPDT group.^18^ A systematic review showed that the use of aPDT could improve periodontal outcomes in the treatment of periodontal and peri-implant diseases and also for HIV-positive patients with periodontitis.^19-26^ However, three meta-analyses^27-29^ and several clinical trials^30,31^ reported no particular advantage for aPDT plus SRP for the treatment of periodontitis.

Considering the existence of conflicting reports and lack of the evidence for comparing the effect of photodynamic therapy (aPDT) sessions on the clinical outcomes, this study sought to compare the effect of adjunctive one to two sessions of aPDT with conventional SRP on the clinical parameters of periodontal health and *F. nucleatum* counts in periodontal pockets of patients with chronic periodontitis.

## Methods

### Patient Selection

The study protocol was approved in the Ethics Committee of Shahid Beheshti Dental School. This split-mouth clinical trial was conducted on 20 patients (9 females and 11 males) with moderate to severe chronic periodontitis, who were selected from the patients presenting to the Periodontics Department of Shahid Beheshti Dental School, using convenience sampling. The inclusion criteria were general health, presence of a minimum of two teeth with a probing depth of ≥5 mm in each quadrant, and CAL≥3 mm. The exclusion criteria were systemic diseases affecting the outcomes of periodontal treatment, a history of periodontal therapy in the past six months, use of antibiotics in the past six months, smoking, pregnancy or nursing, use of non-steroidal anti-inflammatory drugs, and the presence of dental implants. The treatment protocol was thoroughly explained to patients, and written informed consent was obtained.

### Treatment

A thorough clinical examination was carried out in all the patients, and six areas in each tooth, except for the third molars, were examined. After oral hygiene instructions, SRP was performed for all the patients by a trained dentist, using hand and ultrasonic instruments. Random allocation of quadrants to the four treatment groups was carried out with the toss of a coin. Group 1 was subjected to SRP at baseline. Group 2 was subjected to SRP at baseline and one month later. Group 3 was subjected to SRP plus aPDT at baseline, and group 4 was subjected to SRP plus aPDT at baseline and one month. Baseline or time zero refers to the time of initiation of the first phase of periodontal therapy until completion of root debridement. The one-month time interval refers to one month after completion of the debridement of the first phase. The patients were not aware of the treatment modality assigned to each quadrant. Light irradiation was performed using Fotosan 630 LED light-curing unit (CMS Dental, Denmark) at 620‒640-nm wavelength with an output power of 2000 to 4000 µW/cm^2^ with 1.2 mA. Toluidine blue O (TBO) photosensitizer with 0.1 mg/mL concentration (FotoSan Agent Medium Viscosity, FotoSan; CMS Dental, Copenhagen, Denmark) was also used. All the phases of treatment were carried out according to the manufacturer’s instructions. In groups 3 and 4, the tip of a syringe containing TBO was positioned at the opening of the periodontal pocket, and TBO was injected into the pocket until it was filled. Three minutes after the injection of TBO into the pocket, excess TBO was rinsed off using sterile saline, and the Perio-tip of the device inserted into the sulcus was activated for light irradiation. The duration of light irradiation was 10 seconds for pockets shallower than 5 mm and 20 seconds for pockets deeper than 5 mm.

All the clinical parameters were measured by the same calibrated clinician blinded to the type of treatment allocated to each quadrant. Clinical parameters, including PD, CAL gain, and bleeding on probing (BoP), were measured in five patients who had not been enrolled in the study in two sessions (twice) with a 48-hour interval. Calibration was ensured if the percentage of agreement between the baseline and 48-hour measurements was >90%. The clinical parameters were measured in patients at baseline, one month and three months; the PI was measured to assess the oral hygiene of patients; the PD was defined and measured as the distance from the free gingival margin to the depth of the periodontal pocket. The CAL was defined and measured as the distance from the cementoenamel junction to the depth of the pocket. Williams probe (Hu-Friedy, Chicago, IL, USA) was used to measure the PD, BoP, and CAL. Change in the PD was the primary outcome measure, and the change in CAL was considered as the secondary outcome measure (secondary treatment result) in patients.

### Statistical Analysis

Considering the quantitative nature and normal distribution of PD and CAL gain as well as microbial counts, ANOVA was used for the comparison of these variables between the groups. Pairwise comparisons were carried out using t-test. For CAL gain, since non-normal distribution was also noted, Kruskal-Wallis and Mann-Whitney U tests were applied. For intra-group comparisons at different time intervals, quantitative data with normal distribution were analyzed by ANOVA, while quantitative data with non-normal distribution were analyzed by Friedman test.

## Results

### Clinical Findings

Table 1 presents the means and standard deviations of PI, PD, and CAL gain in patients in the four groups at baseline and one and three months after treatment. At one- and three-month postoperative intervals, a significant reduction in clinical parameters of periodontal health was noted in all the groups compared to baseline. At baseline, no significant difference was noted between the groups in clinical parameters. At one month, no significant difference was noted in the PD between the groups. However, at three months, the difference in the PD between groups 1 and 3 was statistically significant (P=0.014). The differences in the CAL gain between groups 2 and 4 at one month (P=0.016), between groups 3 and 4 at three months (P=0.04), and between groups 2 and 4 at three months (P=0.001) were statistically significant (Table 1).

**Table 1:** The means and standard deviations of PD and CAL in the four study groups and the P-values for their comparisons at different time intervals

**Groups**	**Time**	**PD** Mean ± SD* (mm)	**CAL** **Mean ± SD (mm)**
**Group 1** **(SRP)**	Baseline	2.9±1.26	2.29±1.91
	At one month	1.87±1.11	1.35±1.13
	P-value	0.001	0.001
	At three months	1.69±0.94	1.58±1.19
	P-value	0.001	0.001
**Group 2** **(SRP twice with one month interval)**	Baseline	3.09±1.26	2.36±1.52
	At one month	1.96±0.98	1.75±1.02
	P-value	0.001	0.001
	At three months	1.61±0.74	1.59±1.02
	P-value	0.001	0.001
**Group 3** **(SRP + aPDT)**	Baseline	3.23±1.4	2.13±1.63
	At one month	1.97±1.07	1.62±1.13
	P-value	0.001	0.001
	At three months	1.72±0.93	1.55±1.14
	P-value	0.001	0.001
**Group 4** **(SRP + aPDT twice with one month interval)**	Baseline	3.23±1.28	2.51±1.92
	At one month	1.88±0.92	2.00±1.40
	P-value	0.001	0.001
	At three months	1.60±0.80	1.80±1.32
	P-value	0.001	0.001
**P-value for inter-group comparison**	Baseline	>0.05	>0.05
	At one month	>0.05	0.016 (4 & 2)
	At three months	0.014 (3 & 1)	0.040 (4 & 3) 0.001 (4 & 2)

*SD: Standard deviation

To make a better comparison between the groups, the tested areas were divided into two groups of PD<4 mm and PD≥4 mm. There were 63, 66, 90, and 75 areas with PD≥4 mm in groups 1 to 4, respectively. Table 2 presents the means and standard deviations of clinical parameters in the four groups at different time intervals in areas with PD≥4 mm. At one and three months postoperative intervals, a significant reduction in the clinical parameters of periodontal health was noted in all the groups compared to baseline (P=0.001). Although changes in the PD were not significantly different between the groups at the three-month postoperative interval, groups 2, 3, and 4 exhibited a greater reduction in PD at one month compared to group 1; this difference was statistically significant (Table 2). There was a significant difference in PD and CAL gain between the groups at the one- and three-month postoperative intervals (Table 2).

**Table 2:** The means and standard deviations of PD and CAL in areas with PD≥4 mm and the P-values

**Clinical parameters**	**Time**	**Group 1** **(SRP)**	**Group 2** **(SRP twice with one month interval)**	**Group 3** **(SRP + aPDT)**	**Group 4** **(SRP + aPDT twice with one month interval)**	**P-value for inter-group comparison**
**PD** **Mean ± SD (mm)**	**Baseline**	5.45±0.82	5.71±0.90	5.63±0.86	3.47±1.29	-
	**At one month**	3.47±1.29	2.93±1.14	2.98±1.37	2.63±1.27	0.02 (1 & 2) 0.02 (1 & 3) 0.01 (1 & 4)
	**At three months**	3.11±1.27	2.70±1.32	2.59±1.28	2.39±1.18	0.13
**CAL** **Mean ± SD (mm)**	**Baseline**	3.13±3.04	2.87±1.56	2.65±1.94	2.20±1.27	-
	**At one month**	2.97±2.20	1.73±0.81	1.98±1.44	2.10±0.31	0.001(1 & 2)
	**At three months**	2.06±1.92	1.48±0.67	1.39±0.90	2.15±1.40	0.001 (3 & 4) 0.002 (3 & 1)

*SD: Standard deviation

In addition, the tested regions were divided into two groups with CAL gain<1 mm and CAL gain≥1 mm. A total of 337, 273, 257, and 306 areas exhibited CAL gain≥1 mm in groups 1 to 4, respectively. Table 3 presents the means and standard deviations of clinical parameters in the four groups at different time intervals in areas with CAL gain≥1 mm. The mean changes in PD and CAL gain were significant in all the groups after treatment (P<0.001). There was a significant difference in PD and CAL gains between the groups at one- and three-month postoperative intervals (Table 3).

**Table 3:** The means and standard deviations of PD and CAL in areas with CAL≥1 mm and the P-values

**Clinical parameters**	**Time**	**Group 1** **(SRP)**	**Group 2** **(SRP twice with one month interval)**	**Group 3** **(SRP + aPDT)**	**Group 4** **(SRP + aPDT twice with one month interval)**	**P-value for inter-group comparison**
**PD** **Mean ± SD (mm)**	**Baseline**	4.20±1.45	4.58±1.65	4.31±1.27	4.62±1.65	-
	**At one month**	2.48±2.26	2.52±1.79	2.79±1.54	2.90±2.03	0.04 (2 & 4)
	**At three months**	2.15±1.54	2.79±1.62	2.57±1.30	2.78±1.75	>0.05
**CAL** **Mean ± SD (mm)**	**Baseline**	3.02±1.42	2.91±1.26	3.23±1.41	3.21±1.28	-
	**At one month**	1.91±1.26	1.73±0.76	0.93±0.80	1.97±1.04	0.039 (1 & 4)
	**At three months**	1.00±0.64	1.55±0.73	1.60±0.75	1.58±0.80	0.000 (1 & 2) 0.005 (1 & 3) 0.000 (1 & 4)

*SD: Standard deviation

### Microbial Findings

Sixty-eight areas were evaluated in terms of microbial counts. The values presented are in fact the light intensities of gel bands, indicating the amount of DNA present in the respective band, which is also equal to the number of bacteria in the respective samples. A total of 17, 18, 18, and 15 regions were evaluated in groups 1 to 4, respectively. Table 4 presents the means and standard deviations of band intensities for *F. nucleatum* at baseline and at the three-month postoperative interval as well as the P-values for inter-group comparisons at different time intervals. Based on the results of Kolmogorov-Smirnov test, the data were distributed normally. The results of ANOVA showed no significant difference in the mean values between the four groups at any time interval (P>0.05); however, a greater reduction in *F. nucleatum* counts was noted in group 3 (Table 4).

The results showed that all the treatment modalities significantly decreased *F. nucleatum* counts at the depth of pockets.

**Table 4:** The band intensities of samples in the four study groups at different time intervals and P-values for inter-group comparisons

**Group**	**Band intensity at baseline**	**Band intensity at three months**	**P-value**
**Group 1** **(SRP)**	45355.88±9736.47	41653.00±13896.55	0.001
**Group 2** **(SRP twice with one month interval)**	47249.61±12230	41679.16±11656.53	0.022
**Group 3** **(SRP + aPDT)**	42945.94±9150.43	42215.50±11485.11	0.003
**Group 4** **(SRP + aPDT twice with one month interval)**	42909.07±10780	39222.47±10376.38	0.001

## Discussion

The main objective of this double-blind, split-mouth clinical trial was to assess the effect of aPDT as an adjunct to SRP on patients with moderate to severe chronic periodontitis. The results showed that all the treatment modalities tested in this study caused a significant reduction in clinical parameters. A combination of aPDT and SRP caused a greater reduction in PD compared to SRP alone in one month. However, at three months, the four treatment groups were equally effective in the reduction of PD. The CAL gain was higher in the group subjected to aPDT once compared to group 4 subjected to aPDT twice. All the groups exhibited a significant reduction in bacterial counts after treatment. Although the four groups were not significantly different in this regard, group 3 (SRP + aPDT) exhibited a greater reduction in bacterial counts.

Pourabbas et al^32^ and Bassir et al^33^ showed that clinical parameters, such as BoP, PD, and CAL gain, improved in patients with chronic periodontitis after aPDT; however, no significant difference was noted between the groups. These results were consistent with our findings. In a study by Ahad et al^34^ the gingival bleeding index experienced a significant reduction in aPDT group compared to SRP at three months after treatment, but PD and CAL gain were not significantly different between the two groups. Their findings were also consistent with ours. The results of review studies by Sgolastra et al^28^ and Xue et al^35^ showed that the use of aPDT as an adjunct to SRP in patients with chronic periodontitis had short-term benefits, and sufficient evidence regarding its long-term efficacy did not exist. Abduljabbar et al^28^ showed that aPDT was effective in the treatment of chronic periodontitis in diabetes mellitus subjects. However, concerning the effect of aPDT, as an adjunct, as compared to SRP alone, on the clinical signs of chronic periodontitis in diabetes mellitus subjects, no difference could be observed for PD, CAL gain, and HbA1c levels. Sculean et al^36^ reported that aPDT, along with SRP, improved BoP and PD in the short-term. Also, Mongardini et al^37^ and Corrêa et al^38^ reported that aPDT + SRP significantly improved PD, CAL gain, and BoP. Martins et al^23^ reported that in deep periodontal pockets (PD>5 mm), aPDT plus flap debridement resulted in a significantly higher PPD reduction compared to flap debridement alone.^23^ Betsy et al^39^ demonstrated that patients experienced a reduction in gingival bleeding and pain during mastication as well as halitosis after one session of aPDT. However, Romeo et al^40^ reported no significant reduction in PD, crestal bone loss or BoP 12 months after aPDT along with mechanical debridement compared to debridement alone for the treatment of peri-implantitis.^40^ da Cruz et al^41^ reported that aPDT as an adjunct to SRP did not make a difference in terms of periodontal outcomes. Several systematic reviews have demonstrated that the efficacy of aPDT, as an adjunct to SRP, for oral decontamination,^42^ the bactericidal effect,^43^ and as compared with adjunctive antibiotic therapy^44^ remains unclear and debatable. Al-Hamoudi et al^45^ reported that it remains debatable whether aPDT, as an adjunct to SRP, is effective in improving clinical, microbiological, and immunological outcomes compared to SRP alone in type 2 diabetes mellitus and smokers with chronic periodontitis. Discrepancies in the results of studies might be explained by the differences in the type of photosensitizers, light sources, and irradiation parameters. Although the wavelength of light used in different studies is often similar, irradiation parameters and types of photosensitizers used are widely variable. Considering the discrepancies in the results of studies, it appears that the parameters mentioned above affect the treatment outcomes. At present, there is no consensus on a uniform protocol in terms of the frequency of aPDT.

On the other hand, the low-level laser has been the light source used in most previous studies, and studies on the efficacy of aPDT with LED light sources are limited. LED light sources have advantages, such as easy maintenance and affordability. On the other hand, LED light sources do not produce high-intensity monochromatic light; instead, they generate full-spectrum high-intensity light.^46^ Full-spectrum light sources emit light in TBO light absorption spectrum and thus, enhance aPDT.^47^ Giannelli et al^48^ showed that aPDT with LED light inactivated lipopolysaccharides in *P. gingivalis* attached to titanium surfaces. Dilsiz et al^49^ showed that the use of potassium-titanyl-phosphate (KTP) laser along with SRP caused a greater reduction in PD and greater improvement in CAL gain compared to aPDT along with SRP and SRP alone. Birang et al^50^ reported a greater improvement in CAL gain in the laser therapy and aPDT group compared to the SRP group; PD in the laser therapy group exhibited a greater reduction compared to other groups.

Several photosensitizers have been suggested with adequate efficacy against microorganisms without destroying the host tissue.^15^ Limited in vivo studies have evaluated the efficacy of aPDT with TBO. First, in vitro studies showed that the use of a photosensitizer that absorbs a particular wavelength could decrease oral bacterial counts.^51^ In vivo studies later showed that light sensitization of periodontopathogens present in the biofilm could result in their elimination without damaging the host tissues.^16^ Several studies have shown that gram-positive bacteria are more sensitive to aPDT, while gram-negative bacteria are resistant to many photosensitizers used in aPDT.^9^ Wilson et al^52^ showed that subgingival application of methylene blue in areas with chronic periodontitis had greater efficacy in the reduction of microbial parameters compared to control areas treated with sterile water. Another study showed that the use of TBO alone caused a significant reduction in the counts of periodontopathogens on contaminated implant surfaces.^53^ In the current study, TBO was used as a photosensitizer since it can more effectively attach to lipopolysaccharides in the cell wall of gram-negative bacteria compared to methylene blue; however, at 660-nm wavelength, methylene blue can have higher resonance. Also, it has been reported that TBO has bactericidal effects.^54^ In the current study, aPDT caused a significant reduction in *F. nucleatum* counts. Although the difference between the groups was not significant in this regard, this reduction was slightly greater in group 3 subjected to aPDT once.

Sculean et al^36^ reported, in a review study, that in seven out of 14 articles evaluated, aPDT caused a significant reduction in the counts of periodontal pathogens in patients with chronic periodontitis; however, in other studies, this reduction was not significant. Corrêa et al^38^ and Mongardini et al^37^ reported that aPDT + SRP caused a significant reduction in *Aggregatibacter actinomycetemcomitans* and the red complex bacteria. Segarra-Vidal et al^55^ reported that aPDT plus SRP caused a significant reduction in *Aggregatibacter actinomycetemcomitans* in moderate to severe chronic periodontitis patients. The discrepancies in the results of different studies and the present might be attributed to the use of different bacterial strains. Carvalho et al^56^ reported that improvements in clinical and microbial parameters were not significantly different between aPDT and control (sham) groups.

Moreover, Talebi et al^57^ demonstrated that aPDT + SRP did not cause a significant reduction in periodontal pathogens compared to SRP alone. Their findings were consistent with those of the present study. Vohra et al^58^ reported that aPDT, along with SRP, was effective in reducing periodontal parameters in patients with aggressive periodontitis. They showed that aPDT and SRP were effective against several bacterial species, and SRP was more effective in the reduction of red complex periodontopathogens. Their results can justify our findings since, in the present study, none of the treatment groups was superior over the others in the reduction of *F. nucleatum* counts as all the groups received SRP. Also, in the current study, all the patients were not able to maintain ideal oral hygiene since they had poor socioeconomic status. Since all the groups received SRP, and improvements in the clinical parameters were greater in group 1, favorable results of SRP might have masked the efficacy of the adjunct treatment.

Within the limitations of this study (evaluation of only two clinical parameters and one bacterial strain), the results showed that the mean CAL and PD significantly decreased in all the groups with no significant difference in this regard among the groups. Also, all four modalities were successful in decreasing the *F. nucleatum* counts, and none of the modalities had any superiority over the others in this respect. However, the bacterial reduction was slightly greater in the group subjected to aPDT once. Future studies with larger sample sizes are required on a greater number of bacterial species. Also, the role of irradiation parameters and different photosensitizers in the efficacy of aPDT must be evaluated in future studies. Increasing the duration of the follow-up of patients to 12 months and comparison of different treatment protocols, such as repetition of treatment every three months, can help in the selection of the best modality for non-surgical management of chronic periodontitis.

## Competing Interests

The authors declare no conflict(s) of interest related to the publication of this work.

## Authors’ Contributions

LA: design of the work, MSM: analysis, MM: analysis, TM: interpretation of data for the work, KM: interpretation of data for the work, AR: drafting the work, KM: drafting the work, AM: the acquisition, TH: the acquisition.

## Ethics Approval

All the experiments on human subjects were conducted in accordance with the Declaration of Helsinki and all the procedures were carried out with the adequate understanding and written consent of the subjects. The proposal of the study was approved by the Ethics Committee of Research Department of Shahid Beheshti Dental School, and informed consent was obtained for experimentation with human subjects.
